# Identification of key transcription factors and their functional role involved in *Salmonella typhimurium* infection in chicken using integrated transcriptome analysis and bioinformatics approach

**DOI:** 10.1186/s12864-023-09315-3

**Published:** 2023-04-25

**Authors:** Syed Mudasir Ahmad, Sahar Saleem Bhat, Shaista Shafi, Mashooq Ahmad Dar, Afnan Saleem, Zulfqarul Haq, Nida Farooq, Junaid Nazir, Basharat Bhat

**Affiliations:** 1 Division of Animal Biotechnology, FVSc & AH, Shuhama, Sher-E-Kashmir University of Agricultural Sciences and Technology of Kashmir, Srinagar, 190006 India; 2 Department of Clinical Biochemistry, University of Kashmir, Srinagar, India; 3 Division of Livestock Production and Management, FVSc & AH, Shuhama, Sher-E-Kashmir University of Agricultural Sciences and Technology of Kashmir, Srinagar, 190006 India

**Keywords:** Transcription factors, *Salmonella typhimurium*, Chicken, RNA seq, Bioinformatics

## Abstract

*Salmonella enterica* serovar *typhimurium* is the cause of significant morbidity and mortality worldwide that causes economic losses to poultry and is able to cause infection in humans. Indigenous chicken breeds are a potential source of animal protein and have the added advantage of being disease resistant. An indigenous chicken, *Kashmir favorella* and commercial broiler were selected for understanding the mechanism of disease resistance. Following infection in *Kashmir favorella*, three differentially expressed genes Nuclear Factor Kappa B (NF-κB1), Forkhead Box Protein O3 (FOXO3) and Paired box 5 (Pax5) were identified. FOXO3, a transcriptional activator, is the potential marker of host resistance in *Salmonella* infection. NF-κB1 is an inducible transcription factor which lays the foundation for studying gene network of the innate immune response of Salmonella infection in chicken. Pax5 is essential for differentiation of pre-B cells into mature B cell. The real time PCR analysis showed that in response to *Salmonella* Typhimurium infection a remarkable increase of NF-κB1 (*P*˂0.01)_,_ FOXO3 (*P*˂0.01) gene expression in liver and Pax5 (*P*˂0.01) gene expression in spleen of *Kashmir favorella* was observed*.* The protein–protein interaction (PPI) and protein-TF interaction network by STRINGDB analysis suggests that FOXO3 is a hub gene in the network and is closely related to Salmonella infection along with NF-κB1. All the three differentially expressed genes (NF-κB1, FOXO3 and PaX5) showed their influence on 12 interacting proteins and 16 TFs, where cyclic adenosine monophosphate Response Element Binding protein (CREBBP), erythroblast transformation-specific (ETSI), Tumour-protein 53(TP53I), IKKBK, lymphoid enhancer-binding factor-1 (LEF1), and interferon regulatory factor-4 (IRF4) play role in immune responses. This study shall pave the way for newer strategies for treatment and prevention of Salmonella infection and may help in increasing the innate disease resistance.

**Supplementary Information:**

The online version contains supplementary material available at 10.1186/s12864-023-09315-3.

## Introduction

*Salmonella* is a gram-negative, flagellated, rod shaped, facultative anaerobe, and a food borne pathogen which is commonly associated with contaminated food and water. *Salmonella enterica* accounts for approximately 99% of salmonella infections [1]. It has been detected in cattle, pigs, poultry and food products [2]. *S. enterica* serovar *typhimurium* is zoonotic and the most common cause of human food poisoning (11%) which iscaused by poultry products infected by *S. enterica* serovar Typhimurium also written as S. enterica serovar typhimurium or simply *Salmonella typhimurium.* [3]. *Salmonella typhimurium* can be transferred to humans via undercooked or raw infected food, particularly meat and eggs. Salmonella cause an illness called salmonellosis, and some salmonella types can cause typhoid fever, paratyphoid fever as well as gastroenteritis. The lack of an effective immunoprophylactic strategy warrants the need for strict biosecurity in addition to proper cooking and cleaning of food. A So, analysis on *Salmonella typhimurium* infection is highly looked-for, from the perspective of both human and animal health.

Transcription factors play an important role in response to bacterial, viral, and fungal infections, and have been reported as differentially expressed genes involved in disease resistance [4]. Over the last few years, genetic selection of birds is considered to be a systematic and continuous way to control *Salmonella* infections [5]. Selecting chicken that are genetically resistant to *Salmonella* infection offers an alternative environment-friendly control measure. Jammu and Kashmir state in northern India is home to the native breed of chicken known as the Kashmir favorella. It is raised primarily for the production of meat and eggs, but is also regarded as a significant source of animal protein [6]. This native breed is well adapted to the local climatic conditions, feed, and stress management. In addition to this, this breed also has a high disease resistance [7]. In this study, the differentially expressed transcriptional factors NF-κB1, FOXO3 and Pax5 in liver and spleen were examined. After infecting chicken with *Salmonella* Typhimurium, pathogen was first found at the intestinal tract followed by liver and spleen [8]. *Salmonella enterica* can quickly enter the bloodstream, colonize the internal organs including liver, spleen and heart. In this paper identification of immune related genes (NF-κB1, FOXO3 and Pax5) was done. They confer resistance to infection and may have an impact on potential attempts to control *salmonellosis* in chicken and subsequently, help in preventing human *salmonellosis*. These findings will provide newer approaches to develop strategies for *Salmonella typhimurium* prevention and treatment.

## Materials and methods

### Ethics statement

The tissue was collected after approval from Institutional Animal Ethics Committee of Faculty of Veterinary Sciences & Animal Husbandry (SKUAST-Kashmir) ethical standards in animal experimentation (AU/FVSc/PS-57/16021). All the guidelines of the Institutional Animal Ethics Committee of SKUAST Kashmir were strictly followed during all the animal experiments.

#### Experimental birds and Bacterial strain

Susceptible and resistant one day old chicks were initially screened for the presence of any infection. After the initial screening, one day old chicks from *Kashmir favorella*and broiler (Cobb) were procured from Division of Livestock Production and Management, SKUAST-Kashmir,India. The experimental birds were nurtured under standard hygienic conditions at the animal house facility at Faculty of Veterinary Sciences and Animal Husbandry, SKUAST-Kashmir, India. The birds were kept under surveillance daily and had ad-libitum access to feed and water. *Salmonella* Typhimurium strain (ATCC 14,028) from the American Type Culture Collection was procured.

#### Experimental design

A pilot study in *Kashmir favorella* and broiler breeds was carried out where both the breeds were infected at day 4 of age and at the outset were evaluated for disease resistance up to 10 days post infection. The breeds showed optimal differences in clinical symptoms and bacterial loads at day 5 post infection. The birds were divided into two groups i.e., resistant and susceptible groups based on the slight or severity of differences in clinical symptoms, pathological manifestations and bacterial loads. *Kashmir favorella* was found to be resistant while the broilers were found to be susceptible to Salmonella infection based on visible clinical signs and bacterial load. Both the breeds were divided into control and infected groups and were scrutinized for the existence of Salmonella before challenge. Only Salmonella negative birds were taken for the study. After 3 days of habituation, the infected group was challenged orally with 2 × 10^8^ CFU/ml of *S. typhimurium* (14,028) strain. Chicks in the control group were artificially challenged by 1 ml of nutrient broth orally.

The experimental birds were maintained under standard hygienic, temperature and pressure conditions at the animal house facility centre at Faculty of Veterinary Sciences and Animal Husbandry (FVSc & AH), SKUAST-Kashmir, India.. The birds were monitored daily and had ad libitum access to feed and water**.** *Salmonella typhimurium* strain (ATCC 14,028) was obtained from the American Type Culture Collection. The culture was revived in tetrathionate broth (TTB) after incubating at 37 °C for 18 h. The overnight growth from TTB was streaked on MacConkey and Brilliant green agar (BGA) plates, incubated at 37 °C for 24 h and finally examined for typical Salmonella colonies.

### Pilot study

Two experiments were carried out in the current study. A pilot study was conducted to assess the Salmonella resistance and susceptibility between the two chicken breeds (*Kashmir favorella* and commercial broiler). On the basis of the pilot study, the actual experiment was carried out**.**

#### Assessment of Salmonella resistance and susceptibility

For the initial screening of the poultry birds for disease resistance and susceptibility, a pilot study was carried in which one-day old chicks were procured from two poultry breeds i.e. *Kashmir favorella* (*n* = 100) and broiler (Cobb) (*n* = 100). The birds were orally challenged with 2 × 10^8^ CFU/ml of *Salmonella* t*yphimurium* (14,028) strain at day 4 of age and were initially assessed for disease resistance up to 10 days post infection. For confirmation of infection, faecal swabs were taken after 12 h post infection from all the birds and were incubated at 42 °C for 18 h in tetrathionate broth (TTB*)*. Clinical symptoms and gross pathology were also observed. The infection was further confirmed by amplification of Salmonella specific 16S rRNA and *Salmonella* t*yphimurium* specific primers.

The birds were divided into two groups i.e. resistant and susceptible groups based on the differences in clinical symptoms, pathological manifestations and bacterial loads. The chicks that exhibited severe clinical symptoms (progressive weakness, anorexia, diarrhoea and lowering of the head), severe liver pathology and higher bacterial loads in faecal swabs compared with others were identified as challenged susceptible group. Chicks with slight clinical and pathological manifestations and lower bacterial loads were identified as challengedresistant birds. Based on the above clinical symptoms and bacterial loads, *Kashmir favorella* was found to be resistant and broilers were found to be susceptible to Salmonella infection. Day 5 post infection the infected chicken showed optimal differences in clinical symptoms and bacterial loads.

### Actual study

After the initial screening of chicks (resistant and susceptible), one-day-old chicks from both the breeds i.e. *Kashmir favorella* (*n* = 50) and broiler (*n* = 50) procured from the Division of Livestock Production and management, SKUAST-Kashmir-India, were maintained under standard hygienic, temperature and pressure conditions at the animal house facility centre. The chicks were monitored daily and had ad libitum access to feed and water. The chicks from both breeds were divided into control (*n* = 25) and infected groups (*n* = 25) and were screened for the presence of Salmonella before challenge*.* All the chicks were found to be Salmonella negative. After 3 days of acclimatization, the infected group was challenged orally with 2 × 10^8^ CFU/ml of *Salmonella* Typhimurium (14,028) strain. Chicks in the control group were mock challenged and were given 1 ml of nutrient broth orally.

#### Sample collection

The birds were euthanized with CO_2_ and tissue samples (liver and spleen) were taken from twelve infected chicks (resistant 6 and susceptible 6) at day 5 post infection and processed for pathological studies, bacterial enumeration and RNA isolation. Six respective controls were taken from each group. The selection of the chicks for RNA sequencing was based on the highest clinical score, bacterial counts, gross pathology, and lesion scores in their respective groups. The clinical scores were recorded twice daily following points-based scoring system [9].

### RNA-Sequencing data

For global identification of genes and transcription factorss interacting with NF-κB1, FOXO3 and PAX5, we utilized RNA-Sequencing data from our previously published dataset NCBI GEO ID: GSE168060 [10]. The standard protocols/pipeline was utilized for RNA extraction, cDNA preparation and RNA-Sequencing [10]. Total RNA was extracted using Trizol method (Ambion, USA). RNA quality was assessed by spectrophotometer (ThermoFisher, USA) and integrity was assessed by Bioanalyzer (Agilent, USA). Only those RNA samples whose RIN values ≥ 8 were used to construct libraries. Illumina TruSeq stranded mRNA sample preparation kit was used to generate cDNA libraries following manufacturer’s protocol. 4 µg/sample of total RNA was used. Poly-T attached magnetic beads were used for the purification of poly-A containing mRNA molecules. Once the purification was done, the RNA was fragmented into smaller pieces under high temperature using divalent cations. The RNA fragments thus generated, were used for the preparation of first strand cDNA using enzyme reverse transcriptase and random primers (Illumina, USA). Using DNA polymerase 1 and RNase H, the second strand cDNA was synthesized. DNA fragments (at 3ʹ ends) were adenylated and hybridized by ligating Illumina paired-end adapter and index. Using the Illumina PCR primer cocktail, the cDNA fragments (150 bp) were generated and selectively enriched to prepare the final sequencing paired end cDNA library [9]. The High Throughput Model flow cell on an Illumina HiSeq 2500 platform were used to pool the libraries in equimolar amounts and paired end sequenced by SciGenom, Cochin, Kerala-India.

### Total RNA Isolation and cDNA synthesis

Total RNA was extracted from the tissue samples of liver and spleen by Trizol™ method (Invitrogen, USA) as per manufacturer's protocol. The quantity and quality of isolated RNA was checked with UV–Visible spectrophotometer. RNA samples were run on 1% agarose gel to check the quality of RNA. DNase treatment using DNase1 kit (Sigma, USA) was given to rule out genomic DNA contamination. cDNA synthesis was done with equal concentration of RNA (1.5 μg/μl) in all the samples using Thermo Scientific RevertAid First Strand cDNA Synthesis Kit™ (Lithuania) using oligodT primers following manufacturer protocol.

### Quantitative real-time PCR

Real-time PCR was performed to verify the expression of three key transcription factors (NF-κB 1, FOXO3 and PAX5) which are involved in different immunological pathways. Following the manufacturer’s protocol, total RNA was used to synthesize cDNA using revert Aid cDNA synthesis kit (Thermo Scientific, USA). The primer details are enlisted in (Table 1). All the qPCR reactions were run in replicates using Light Cycler II (Roche). β-actin and GAPDH were used as internal controls. The reaction mixture (20 µl) consisted of 8.4 µl nuclease free water, 0.3 µl of 10 µM each forward and reverse primers, SYBR green master mix (Roche) 10 µl and 70 µg/µl of cDNA. The reactions were amplified by 40 cycles of denaturation at 95 °C for 5 min, annealing at variable temperatures (specific for each gene) for 15 s and extension at 72 °C for 15 s. For the confirmation of product specificity, melting curve analysis was done. The relative mRNA expression was determined by using 2^−∆∆CT^ method [11].

**Table 1:** Primer details

Target	Sequence 5ʹ-3ʹ	Tm	Amplicon size (bp)	Reference
β Actin	F: TGGCATTGCTGACAGGAT R: CTGCTTGCTGATCCACAT	63.1 60.0	160	(He et al., 2013) [12]
GAPDH	F: GTCAGCAATGCATCGTGCA R: GGCATGGACAGTGGTCATAAGA	67.5 66.0	180	(Berndt et al., 2007) [13]
NFKB1	F:GAAGGAATCGTACCGGGAACAACACCACTGGATAT R:TTACTGTCACAAGGCCCTCTGAG	78.2 66.5	131	(Chiang et al., 2009) [14]
FOXO3	F: ACTTCAAGGACAAGGGCGACAACAAC R:GGACGGTGGAAAAGTTGGCAAGGC	65.85 66.1	160	(Chen et al., 2019) [15]
PaX5	F: GTCAGCCACGGCTGCGTCAGCAAAATAC R: ATCATACGGACAAAGGTGCAGCAGCC	78.6 74.8	260	(Nera et al., 2006) [16]

### Network and enrichment analysis

STRINGDB was utilized to identify the possible protein–protein interaction (PPI) interaction network. Two topographical features “*Betweenness between nodes”* and “*Degree”* were used to identify key interacting partners in the PPI network. Higher the quantitative value of a node, the more important is the node in the network. The TF were subjected to functional using *g: Profiler* [a] web server using *Gallus gallus* (chicken) as reference organism.

## Result

### Clinical signs

**Fig. 1:**
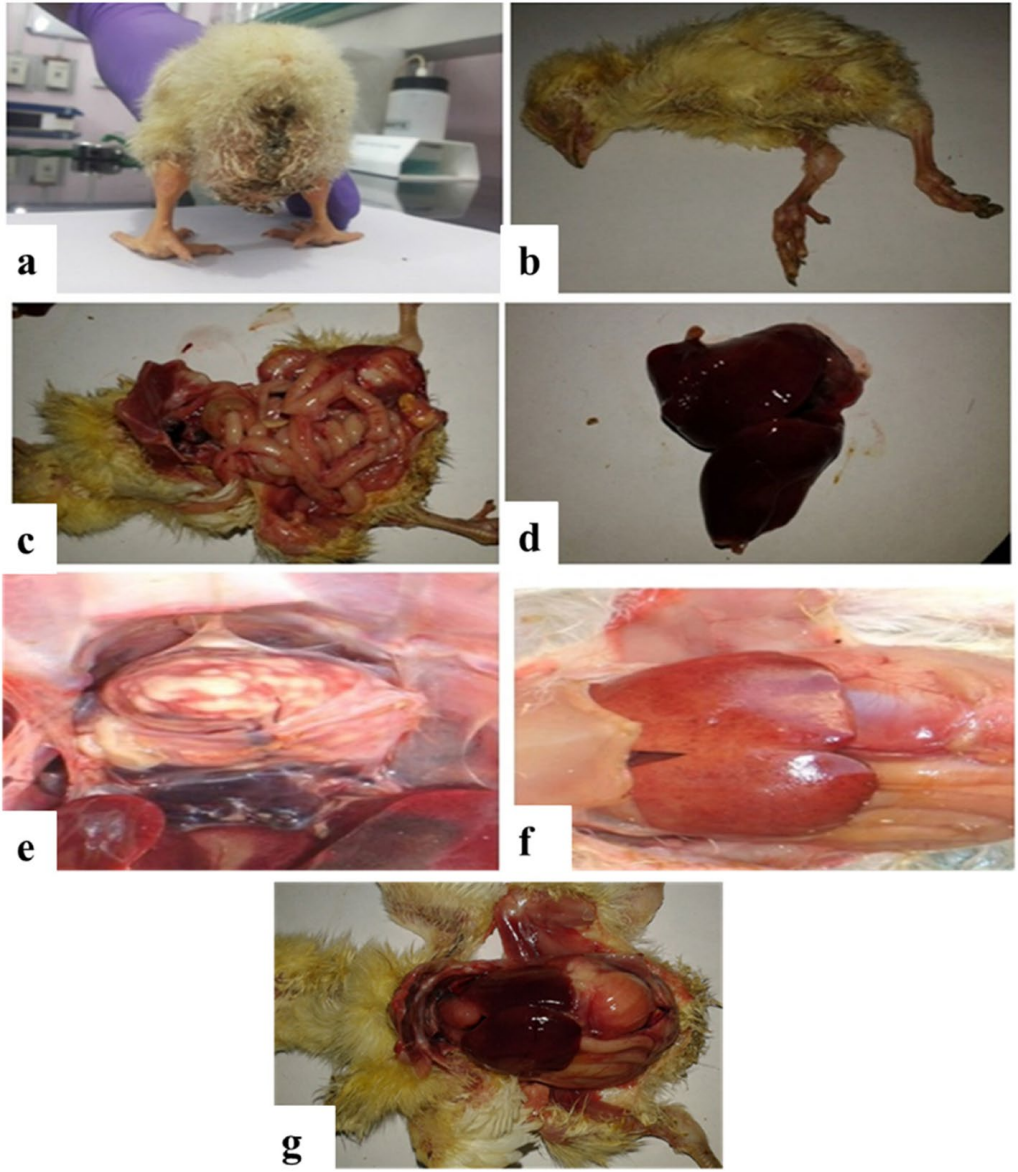
Clinical symptoms and gross pathology following *Salmonella* Typhimurium infection (**a**) Vent paste (**b**) Ruffled feathers (**c**)Intestinal haemorrhages (**d**) Bronze discoloration of liver (**e**) Elevated white nodular lesions on ventricles (**f**) Prominent necrotic foci on liver (**g**) Hepatomegaly

Once the chicks were inoculated with *Salmonella typhimurium*, typical clinical signs of progressive weakness, anorexia, diarrhoea, exhaustion, dullness, ruffled feathers, lowering of head, dropping of wings, inappetence, and reluctance to move with both eyes closed were observed (Fig. 1). For further confirmation of infection, amplification of Salmonella specific 16S rRNA and *Salmonella typhimurium* specific primers was done using PCR.

### Bacterial load

**Fig. 2:**
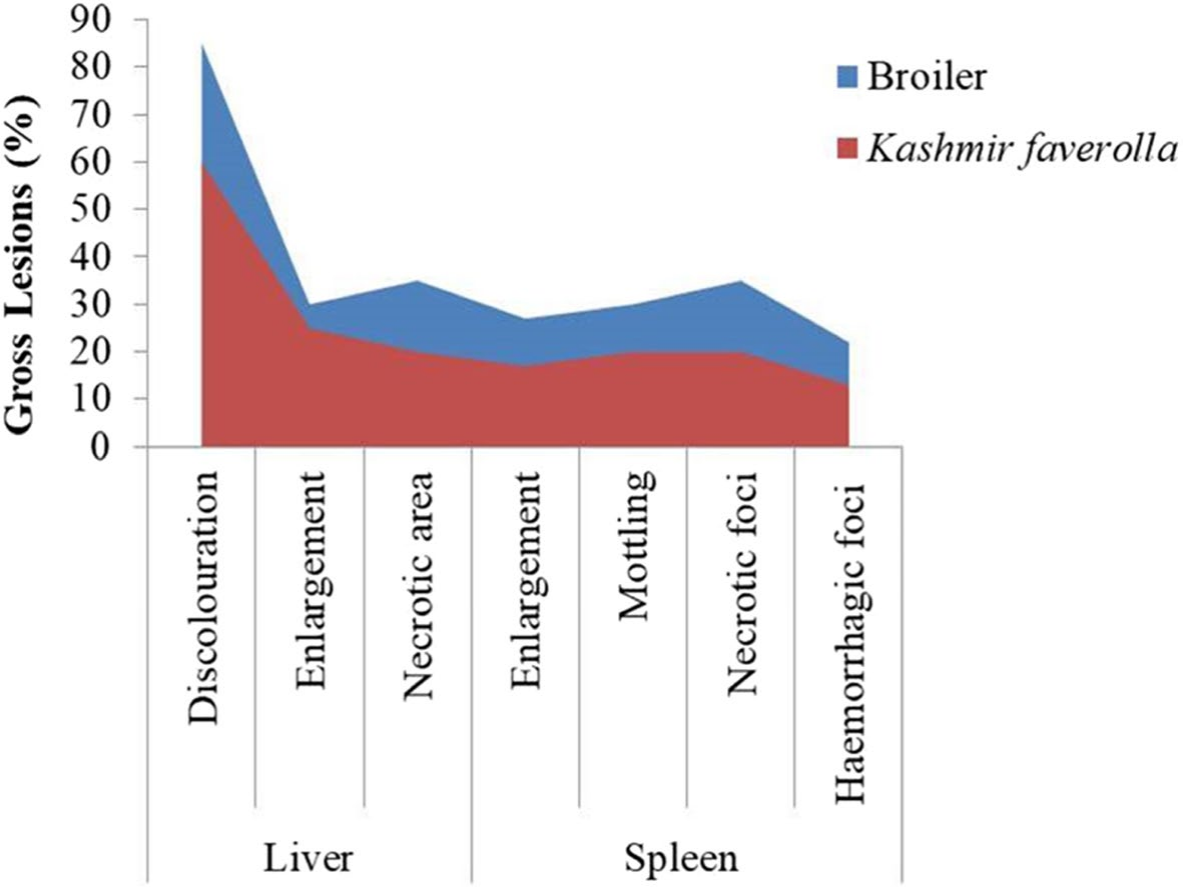
Proportionate distribution of gross lesions in commercial broiler and *Kashmir favorella* observed in case of experimentally induced *Salmonella* Typhimurium infection

**Fig. 3:**
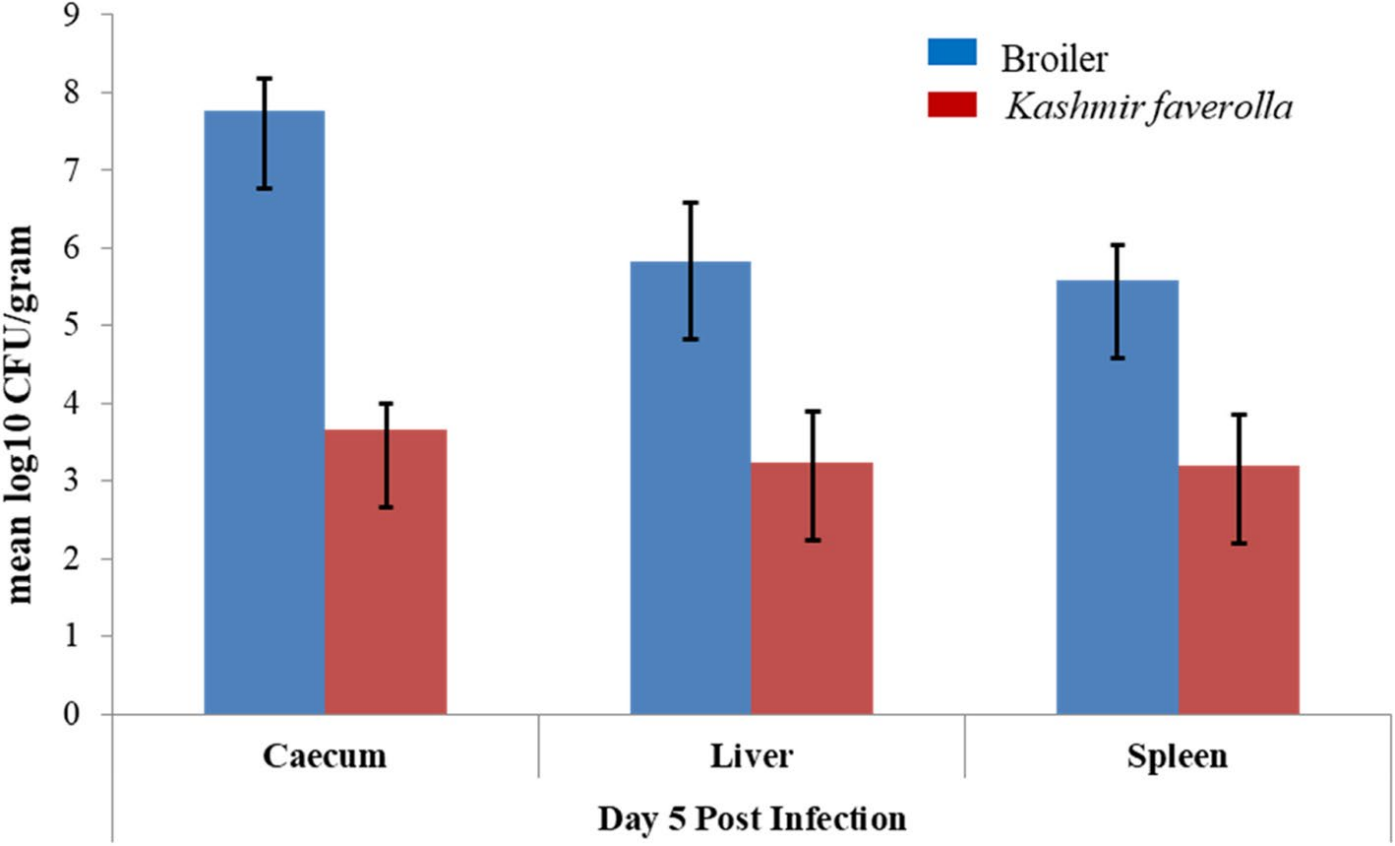
Recovery of *Salmonella* Typhimurium from the caecum, liver and spleen at day 5 post inoculation. The bacterial load is shown as mean log10 CFU/gram

The resistant group i.e. *Kashmiri favorella* displayed mild clinical symptoms with low bacterial load while as the birds in the susceptible group i.e. broiler displayed severe clinical signs with high bacterial load. The greatest differences in clinical symptoms, pathology, and bacterial load were observed on day 5 after inoculation. The resistant birds had minor liver lesions compared to the susceptible birds, which had major necrotic lesions. (Fig. 2 and Fig. 3).

### Gene expression study

#### RNA-seq data

Transcriptome data was cleaned using pipeline described [17]. In this study we performed a multi-group comparison between liver samples (Boiler Liver Infected (A) Vs Boiler Liver Control (B) Vs Kashmiri Liver Control (E)) and spleen samples (Boiler Spleen Infected (C) Vs Boiler Spleen Control (D) Vs Kashmiri Spleen Control (G) Vs Kashmiri Spleen Infected (F) to identify more genes and TFs associated with infection resistance. In accordance with qPCR results, RNA-Sequencing data shows similar trend in the expression of all three TFs in spleen and liver Samples. FOXO3 and NF-κB1 were expressed more in liver and PAX5 in spleen. Fold increase in liver of *Kashmi*r *favorella* liver and spleen is given in Table 2. The PCA plot shows clear separation between samples (Supplementary Fig. 1).

**Table 2:** Top DE genes in spleen and liver samples – RNA-Seq multi group comparison

Gene ID	Gene Symbol	Tissue	*p*-value	q-value
ENSGALG00000011630	GLI2	Spleen	3.83E-09	9.33E-05
ENSGALG00000029576	PDHB	Spleen	8.28E-09	0.000100882
ENSGALG00000015844	SOD1	Spleen	1.79E-08	0.000141526
ENSGALG00000023953	-	Spleen	2.33E-08	0.000141526
ENSGALG00000021525	ISLR	Spleen	2.91E-08	0.000141526
ENSGALG00000016431	PAX5	Spleen	5.11E-08	0.000207264
ENSGALG00000012304	NFKB1	Liver	0.000323	0.016978
ENSGALG00000015297	FOXO3	Liver	0.002906	0.032206

**Fig. 4:**
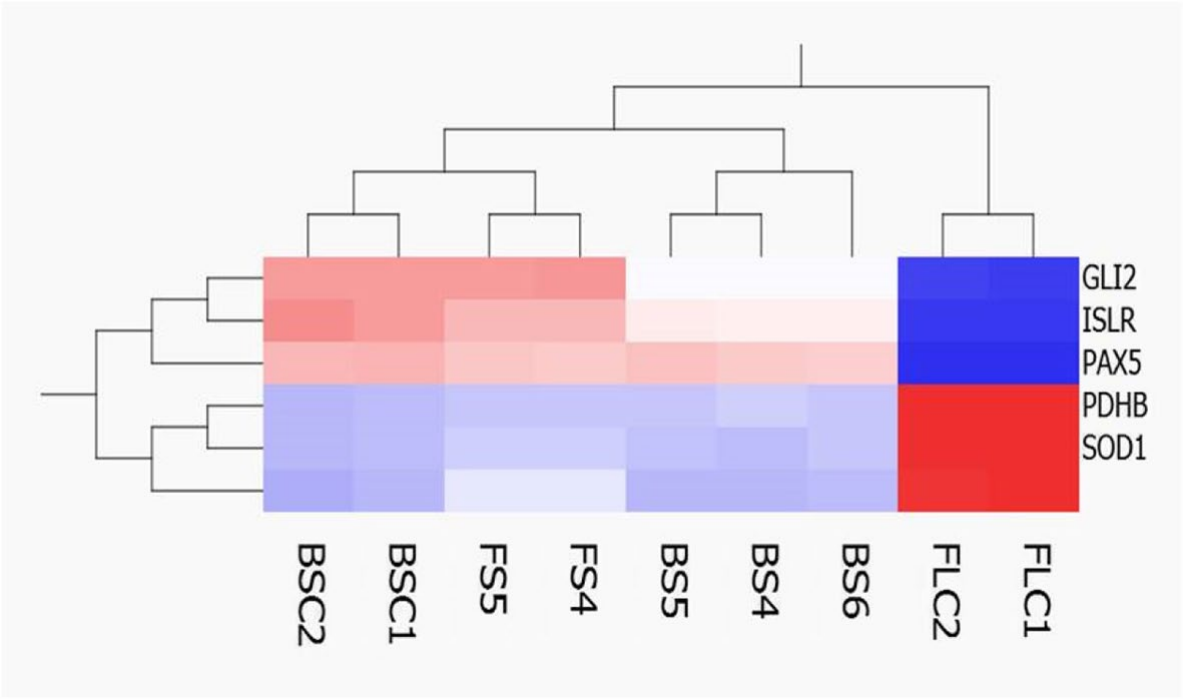
2D-Hierarchical clustering of top DEGs in spleen samples

**Fig. 5:**
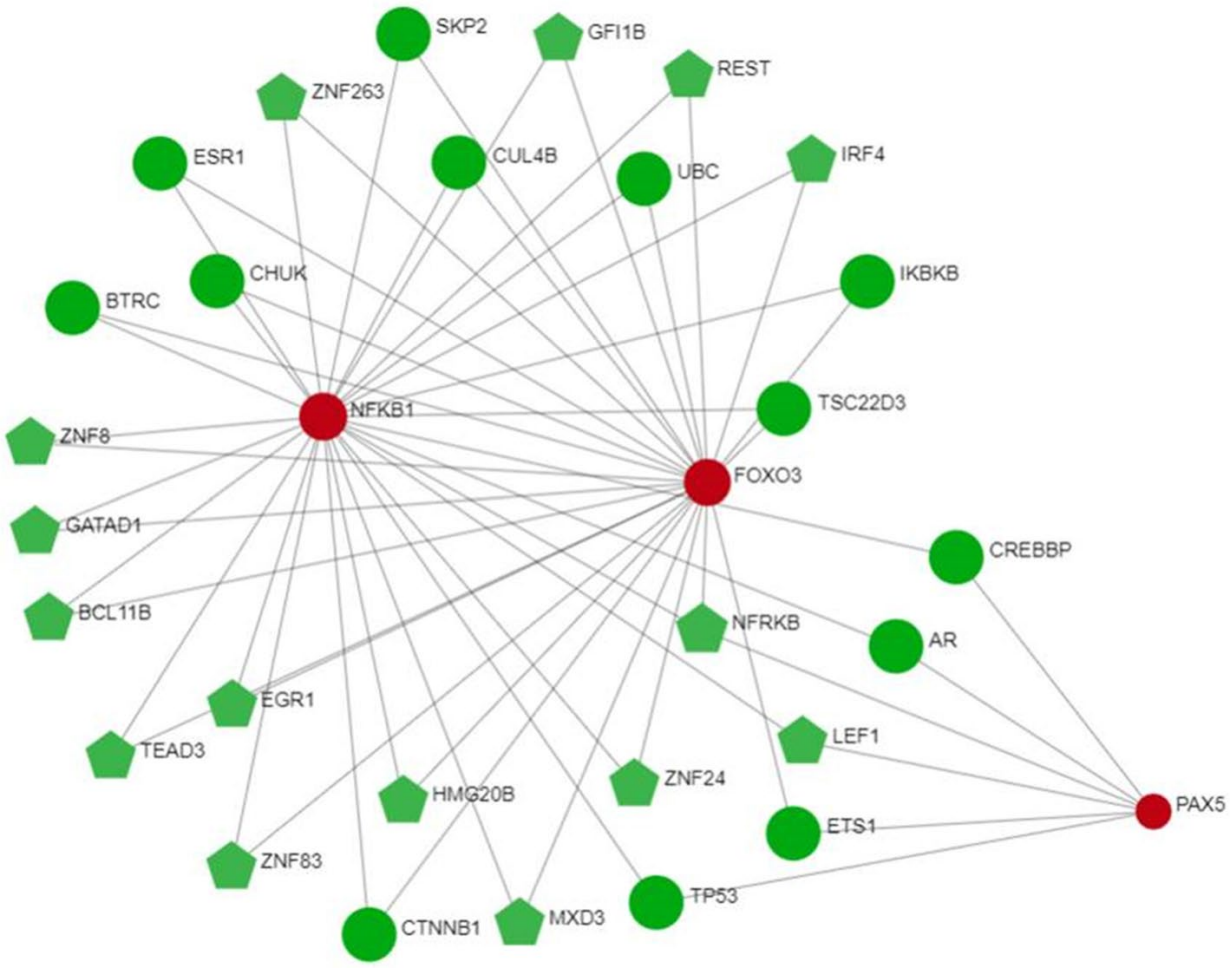
Protein–Protein and Protein-TF interaction network. Pentagon represents TFs, green solid-circles represent interacting proteins and red solid-circles represent TFs of interest in this study. The nodes were selected with degree > 5 and betweenness > 5 threshold

2-Dimensional hierarchical clustering of top DEGs in spleen samples (multi-group comparison) shows the key role of PAX5 in regulating infection resistance in Kashmiri favorella (Fig. 4). The protein–protein and protein-TFs interaction analysis (Fig. 5) suggests that FOXO3 (Degree = 23 after thresholds) was hub gene in the network and closely related in Salmonella infection with NF-κB1, which is in accordance with the RNA-Seq and q-PCR results.

To gain insight into the biological processes and pathways mediated by PAX5, FOXO3 and NF-κB1Gene ontology (GO) and KEGG enrichment analysis was performed. Pathway analyses suggest that NF-κB1 and FOXO3 were involved in *Gallus Gallus* Cellular senescence signalling pathway (FDR adjusted *P*-value = 9.107 × 10^–3^). The GO analysis suggest that all three TFs were involved in molecular function; DNA-binding transcription activator activity (p_adj_ = 2.378 × 10^–3^) and biological process negative regulation of transcription (p_adj_ = 3.064 × 10^–2^). The transcription factors of interest NF-κB1, FOXO3 and PAX5 were significantly upregulated in *Kashmir favorella.* All the three showed their influence on 12 interacting proteins (CREBBP, AR, ETS1, TP53, IKBKB, UBC, CUL4B, SKP2, ESR1, CHUK, BTRC, TSC22D3) and 16 TFs (LEF1, NFRKB, IRF4, REST, GFI1B, GAGAD1, BCL11B, TEADE, EGR1, HMG20, BCTNNB1, MXD3, ZNF263, ZNF8, ZNF83, ZNF24), where CREBBP, ETSI, TP53I, IKKBK, LEF1, IRF4 play role in immune responses as shown in Fig. 5. Furthermore, the differentially expressed genes that were mainly related to immune function, such as TNFRSF11B, SOCS1, IL-18, IL6, IL1β, IL10RB, IKBKB, FOS, and EGR1 were also upregulated in *Kashmir favorella.*

### Real-time quantitative PCR validation of the RNA-seq expression results

**Fig. 6:**
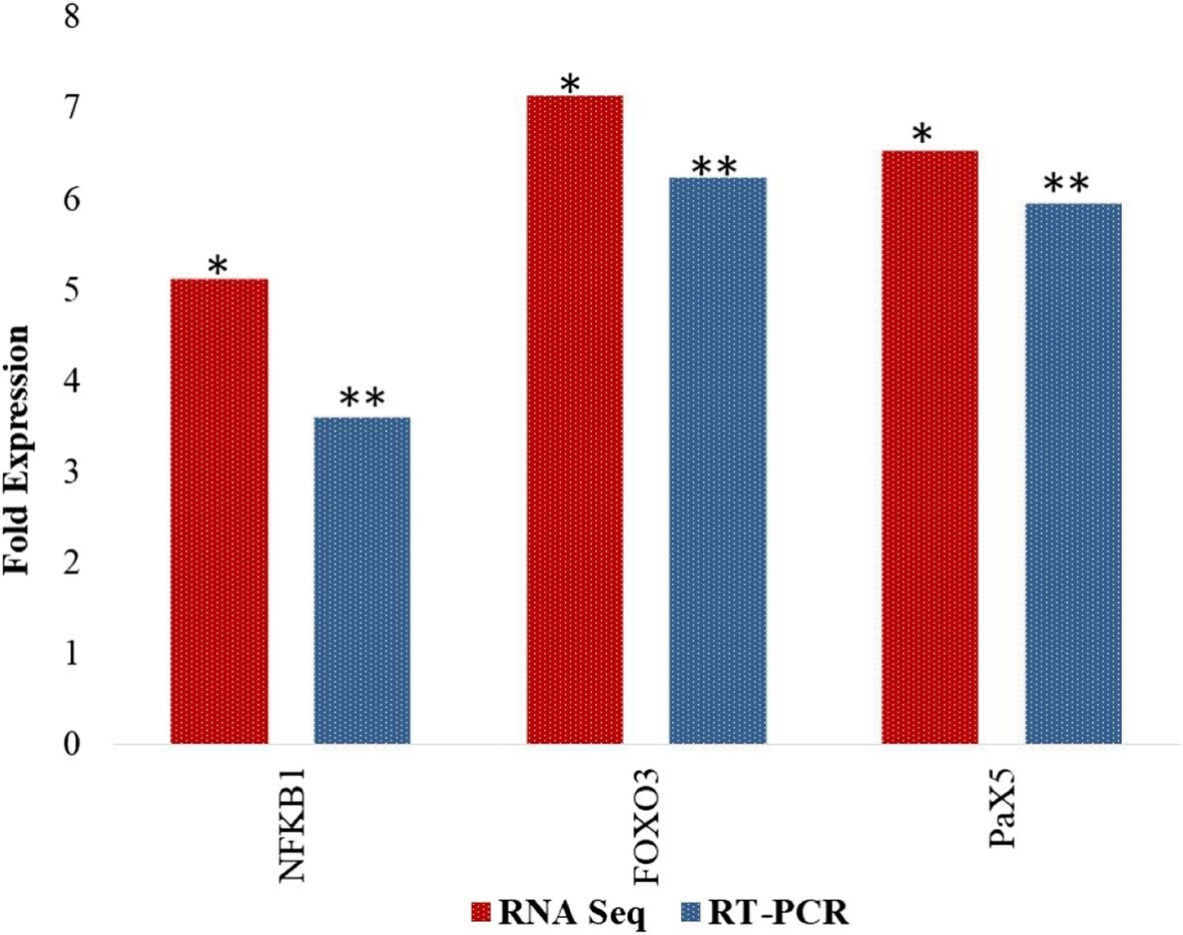
Real time PCR validation of differentially expressed genes. The y-axis represents the log2 fold change DEGs; the x-axis shows the gene names employed for validation. RNA seq:*P < 0.01; RT-qPCR: **P < 0.01

The differentially expressed transcription factors NF-κB1*,* FOXO3 and PAX5 in *Kashmir favorella* were significantly upregulated when compared to commercial broiler where the expression of NF-κB1 was 12.04-fold with *P*˂ 0.01, FOXO3 was 74.54-fold with *P*˂ 0.01 in liver and PAX5 was 61.39-fold with *P*˂0.01 in spleen as given in Fig. 1 and Table 2. Similar trend in the expression of NF-κB1 and PaX5 were identified in RNA seq data. The comparative RNA sequencing expression and qPCR expression is shown (Fig. 6).

## Discussion

The genetic mechanism of disease resistance in poultry against Salmonella infection have been described by several studies. Our study elucidates the genetic basis of disease resistance in *Kashmir favorella* and commercial Broiler against *Salmonella typhimurium* based on the severity of clinical symptoms, bacterial load post infection and the pathological manifestations. Following *Salmonella typhimurium* challenge in chicken liver and spleen, we identified certain transcription factors that played an important role in resistance and susceptibility of two different chicken breeds (Commercial broiler and *Kashmir favorella*). Nuclear Factor kappa B subunit (NF-κB1), Fork head box transcription factor 3 (FOXO3) and Paired box 5 (PaX5), superoxide dismutases (SOD1), Immunoglobulin Superfamily Containing Leucine Rich Repeat (ISLR), Pyruvate Dehydrogenase E1 Subunit Beta (PDHB), Zinc finger protein (GLI2) transcription factors were identified. FOXO3 is involved in a variety of cellular functions including cell-cycle control, DNA damage repair, cell growth and differentiation, response to oxidative stress, and apoptosis [18]. Chicken FOXO3 reportedly shares more than 85.4% identity with humans [19]. The FOXO3 gene controls lymphoid homeostasis in the host immune system [20, 21]. FOXO3 serves as a marker of host resistance to Salmonella infection. In previous studies, JAK-STAT signalling pathway, MAPK-related pathways and cytokine-cytokine receptor interaction were activated significantly in resistant chickens [5]. In our study, mRNA levels of FOXO3 were quantified in liver and spleen of *Kashmir favorella*. FOXO3 is highly upregulated in liver of *Kashmir favorella*. It was found that MAPK pathway, cytokine-cytokine and JAK-STAT pathways are upregulated in *Kashmir favorella*. TNFRSF11B is one of the members of tumor necrosis factor receptor (TNFR) molecular sub-family was also upregulated in *Kashmir favorella* and has role in RANK activation [22]. The ligation of RANK by RANKL plays an important role in the immune response regulating the interactions between T cells and dendritic cells. The JAK-STAT pathway is also activated involving genes IL10RA, IL10RB, SOCS1 positively regulated. The JAK-STAT pathway ensures T and B-cell development [23]. In the present study, various genes involved in the JAK-STAT signalling pathway had increased expression including IL10RB that activate monocytes and neutrophils and SOCS1 that regulates M1 macrophage activation. FOS and IRG1 have role in cell proliferation and differentiation and promote endotoxin tolerance respectively. Also, IL18 has role in IFN-γ production, promote Th1 immunity host defense inflammation (innate and acquired immunity) is highly upregulated in *Kashmir favorella.*

NF-κB1 was more expressed in liver of *Kashmir favorella* than commercial broiler*.* It is a primary regulator of the innate immune response to bacterial invasion. It is activated by MyD88-dependent TLR pathway signalling [24]. It has five related proteins (subunits) that can bind to DNA: p50 (a 50 kDa protein also known as NF-KB1), p52 (also known as NF-B2), p65 (also known as RelA), c-Rel, and RelB. [25]. According to our findings, Salmonella infection causes inflammatory expression of NF-κB genes. NF-κB is the master regulator of the inflammatory response in the complex inflammatory network, serving as both a host defence against pathogens and a cell protector against apoptosis. This occurs as a result of the activation of proinflammatory genes [26]. It is crucial in the activation of chemokines/cytokines (IL18) [27]. The NF-κB1 pathway was successfully activated in Kashmir favorella. Our results showed that NF-κB1 and FOXO3 were upregulated in *Kashmir favorella* play their role in expression of interacting proteins and transcription factors as given in Fig. 6. FOXO3 and NF-κB1 were expressed more in liver, as liver sinusoids monocytes are present in bulk whereas in spleen only red pulp contains monocytes [19, 28]. FOXO3 binds NF-KBRelA, which provides cytoplasmic stability while also inhibiting proinflammatory signalling pathways. NF-κB1 was able to shuttle to the nucleus and turn on proinflammatory target genes (IL-6, IL-12, and IL-15) after FOXO3 expression was silenced [29]. The signalling adapter molecule IKBKB was significantly upregulated in *Kashmir favorella* after Salmonella infection that showed its influence on FOXO3 expression as well. Furthermore, EGR1 and IRF4 were also upregulated. EGR1 plays role in inflammatory responses whereas IRF4 in development of NK cells, B and T cells. Bcl11b is essential for development of CD4^+^ and CD8^+^ T cells [30–32].

The expression of PAX5 (BSAP) was remarkably more in spleen of *Kashmir favorella.* PAX5 expressed more in spleen as it was continuously required for B-cell function [33]. It has 94% similarity with mice PAX5 and is solely expressed from the pro-B to mature B cell stages and is downregulated in the course of terminal differentiation into plasma cells [34]. It is a vital transcription factor in the process of B cell development and intricated in the activation of the chromatin of essential genes involved in B-cell signalling, adhesion, migration, inflammatory and immune responses [35–37]. It plays a principal role in the upregulation of B lymphocyte-induced maturation protein 1 (Blimp-1) and X-box binding protein 1 (XBP-1) [38, 39] obligatory for plasma cell differentiation [3, 40]. Blimp-1 is enough to shut down the B cell gene expression and initiate the development of plasma cells. Apart from this it also plays a significant role in activation of CD19, CD79A and repression of FMS, Notch1, CSF1 receptor [16, 41]. Notch 2 is expressed in liver of *Kashmir favorella.* PAX5 either act as activator or repressor.

Both NF-κB1 and PAX5 play role in the expression of CREBBP, AR, LEF1 and TP53. Lymphoid Enhancer Binding Factor-1 (LEF1) is a Wnt/-catenin signalling pathway mediator that interacts with -catenin to regulate Wnt target gene expression [42, 43]. When the Wnt/-catenin pathway is activated, catenin enters the nucleus and interacts with transcription factors T-cell and lymphoid enhancer factors (TCF/LEF1) to activate and/or repress transcription of specific target genes [44]. LEF1 in chickens is expressed in pre-B and T-cells, T-helper 1 cell differentiation and B-cell proliferation. Cyclic adenosine monophosphate Response Element Binding Protein Binding Protein (CREBBP or CREB) is present in liver of *Kashmir favorella*. The CREB promotes activation and proliferation of T and B cells and differentially regulates Th1, Th2, and Th17 responses [45]. In *Kashmir favorella* IL10, TNF are highly upregulated**.** Tp53 also regulates innate immune and adaptive immune responses. FOXO3 and PaX5 expression also show its effect on ETS1. ETS1 deficiency has a significant impact on B cells, T cells, and NK cells, all of which express ETS1 at high levels under normal physiological conditions [46]. PAX5 and FOXO3 have been shown to interact with ETS1. It is manifested in spleen. The ETS1 transcription factor belongs to the ETS gene family and has been found to be highly conserved throughout evolution (600–700 million years ago). The ETS gene family includes 28 genes in humans (441 amino acids) and 27 genes in mice (440 amino acids). Mice ETS1 has 95% similarity with that of chicken ETS1. Adult humans and chicken show a similar pattern where high expression of ETS1 is found mainly in lymphoid tissues [47, 48]. ETS1 is expressed in B cells (highly expressed in naïve mature B-cells and memory B-cells), T cells, NK cells, and NK T cells [49, 50] and is inducible in other, non-lymphoid cell types in response to certain stimuli [51].

SOD1 in presence of copper and zinc catalyse the transfer of superoxide anions into less dangerous free radical (hydrogen peroxide). It decreases ROS formation [52]. It has antibacterial and anti-fungal activity. SOD1 also show its effect on NF-κB and p38 pathways involved in influenza A virus pathogenesis and pro inflammatory responses. SOD1 overexpression significantly attenuates transcription of several pro inflammatory cytokines like CCL2, IL6, TNFα, IL-8 [53]. ISLR is an immunoglobulin superfamily containing leucine rich repeats that stabilizes Wnt signalling and regulates muscle atrophy (skeletal muscles) via IGF1-PI3K/Akt-FOXO signalling pathway [54] PDHB plays role in metabolic processes TCA cycle, gluconeogenesis acetyl CoA synthesis and canonical pathways [55]. GLI-2 is a zinc finger protein which acts as transcriptional activator in hedgehog (hh) signalling. Expression of GLI-2 has an effect on activation of NF-κB pathway as well as cell apoptosis. Upregulation of GLI-2 decreases the proinflammatory cytokines, interleukin-6, IFN-γ, FASL whereas increases expression IL-10 [56].

## Conclusion

Based on our RNA Seq data, we discovered that *Kashmir favorella* was more resistant to *Salmonella typhimurium* infection than commercial broiler. A consistency in gross pathology, histopathology and lesion score with bacterial load in liver and spleen was found.

Transcriptome profiling of the liver and spleen in two chicken breeds/strains i.e., *Kashmir favorella* and commercial broiler manifest that *Kashmir favorella* shows a significant improvement in the majority of the genes and signalling pathways involved in protecting the host from bacterial infections. The differentially expressed genes NF-κB1, FOXO3 and PAX5 are involved in defending the host against bacterial infections were significantly enriched in the indigenous breed *Kashmir favorella*. Also, the genes that are found to be involved in immune response and disease tolerance were differentially expressed in *Kashmir favorella*. The protein–protein interaction (PPI) and protein -TF interaction networks by STRINGDB analysis suggests that FOXO3 was hub gene in the network and closely related in Salmonella infection with NF-κB1. By using model animals like chicken, these findings will help us better understand the phenomenon of disease resistance and susceptibility to other bacterial and viral infections through pathogen-induced genes. In addition, this research will be useful for developing fresh plans and methods for preventing and treating Salmonella infections. It may also increase innate disease resistance through genetic selection in poultry, which would lower the risk of human Salmonellosis.

## Supplementary Information

**Additional file 1:** **Supplementary figure 1.** The PCA plot shows between different samples. This plot shows clear separation between samples. **Supplementary table 1.** Downloaded RNA-Seq data from Mashooq et al 2022 UR_Genomics_submission number (NCBI Accession number GSE 168060).

## Data Availability

The sequencing data is available in NCBI under accession number GSE168060.
