# Supplementary material for: Identification of key transcription factors and their functional role involved in Salmonella typhimurium infection in chicken using integrated transcriptome analysis and bioinformatics approach
Source: BMC Genomics. 2023 Apr 25;24:214. doi: 10.1186/s12864-023-09315-3 (PMC10127038; doi:10.1186/s12864-023-09315-3)
Supplement: Supplementary file 1 — Additional file 1: Supplementary figure 1. The PCA plot shows between different samples. This plot shows clear separation between samples. Supplementary table 1. Downloaded RNA-Seq data from Mashooq et al 2022 UR_Genomics_submission number (NCBI Accession number GSE 168060). [file 12864_2023_9315_MOESM1_ESM.docx]

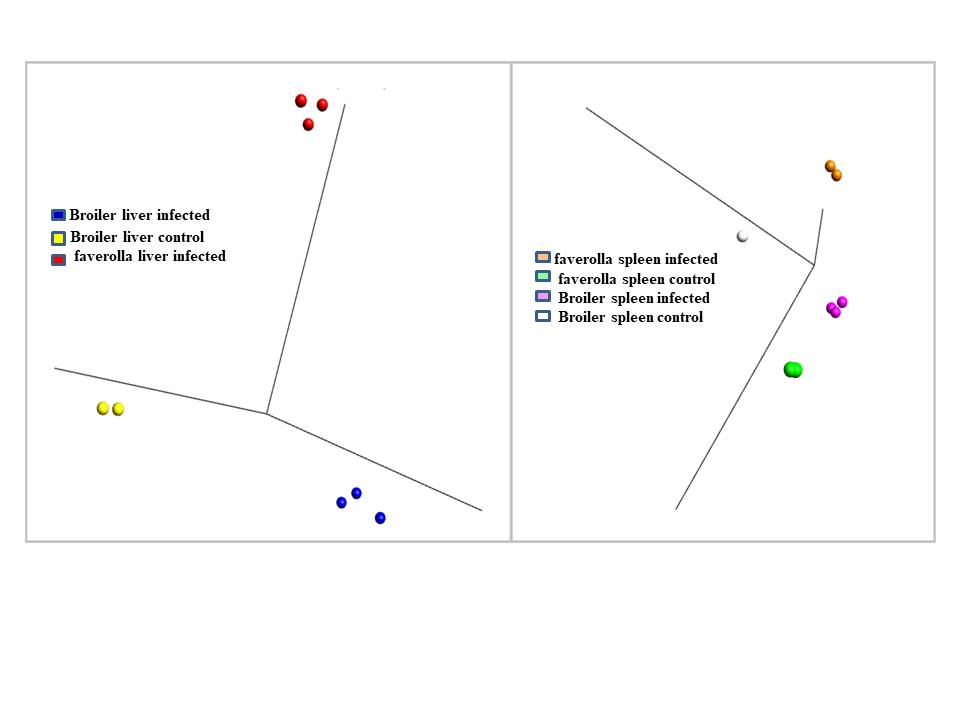


**Supplementary figure 1:** The PCA plot shows between different samples. This plot shows clear separation between samples

**Supplementary table 1. Downloaded RNA-Seq data from** Mashooq et al 2022 UR_Genomics_submission number **(NCBI Accession number GSE 168060).**

| Group | Filename | Group |
| --- | --- | --- |
| A | BL1 | Boiler Liver Replicate 1 |
| A | BL2 | Boiler Liver Replicate 2 |
| A | BL3 | Boiler Liver Replicate 3 |
| B | BLC1 | Boiler Liver Control Replicate 1 |
| B | BLC2 | Boiler Liver Control Replicate 2 |
| C | BS4 | Boiler Spleen Replicate 1 |
| C | BS5 | Boiler Spleen Replicate 2 |
| C | BS6 | Boiler Spleen Replicate 3 |
| D | BSC1 | Boiler Spleen Control Replicate 1 |
| D | BSC2 | Boiler Spleen Control Replicate 2 |
| E | FL1 | Kashmiri Liver Replicate 1 |
| E | FL2 | Kashmiri Liver Replicate 2 |
| E | FL3 | Kashmiri Liver Replicate 3 |
| F | FS4 | Kashmiri Spleen Replicate 1 |
| F | FS5 | Kashmiri Spleen Replicate 2 |
| G | FSC1 | Kashmiri Spleen Control Replicate 1 |
| G | FSC2 | Kashmiri Spleen Control Replicate 2 |
